# From simulation to application: enhancing preclinical evaluation of dissolvable microarray patches through PBPK modelling

**DOI:** 10.1007/s13346-025-01974-x

**Published:** 2025-10-10

**Authors:** Maja Railic, Wilhelmus E. A. de Witte, Stephan Schaller, Sarah Toluwanimi Agboola, Ziad Sartawi, Waleed Faisal, Mohamed Elkhashab, Abina Crean, Sonja Vucen

**Affiliations:** 1 SSPC, Research Ireland Centre for Pharmaceuticals, School of Pharmacy, University College Cork, Cork, T12 K8AF Ireland; 2 ESQlabs GmbH, Am Sportplatz 7, Saterland, 26683 Germany; 3 School of Pharmacy, University College Cork, Cork, T12 K8AF Ireland; 4 ArrayPatch Ltd., Euro Business Park, Little Island, Cork, T45 FX94 Ireland

**Keywords:** Dissolvable microarray patches, Preclinical evaluation, PBPK modelling, Clinical translation

## Abstract

Dissolvable microarray patches (MAP) represent a promising drug delivery platform; however, the absence of standardised protocols for their preclinical evaluation poses a significant barrier to regulatory approval and clinical translation. Physiologically Based Pharmacokinetic (PBPK) modelling is a powerful tool for predicting drug kinetics following MAP application, addressing key challenges associated with in vitro and in vivo studies such as experimental variability, complex study design, and data extrapolating across different populations. However, adapting PBPK models for dissolvable MAP is inherently complex due to the interplay between microneedle geometry, drug release kinetics, and skin physiology. In this study, an existing dermal PBPK model in MoBi^®^ was optimised for dissolvable MAP by incorporating microneedle geometry and in vitro release profiles of MAP formulations containing the antihistamines loratadine (LOR) and chlorpheniramine maleate (CPM), as well as the antifungal drug itraconazole (ITZ). Model refinement involved systematically optimising input parameters related to skin thickness and drug-skin diffusion, partitioning and binding, to enhance predictive accuracy. Validation was performed using in vitro permeation testing with porcine skin for CPM and LOR MAP, alongside in vivo preclinical studies in pigs for ITZ MAP. The optimised model demonstrated robust predictive performance across the diverse drug molecules and experimental conditions investigated, highlighting its value as a powerful tool to accelerate preclinical MAP development.

**Graphical abstract:**

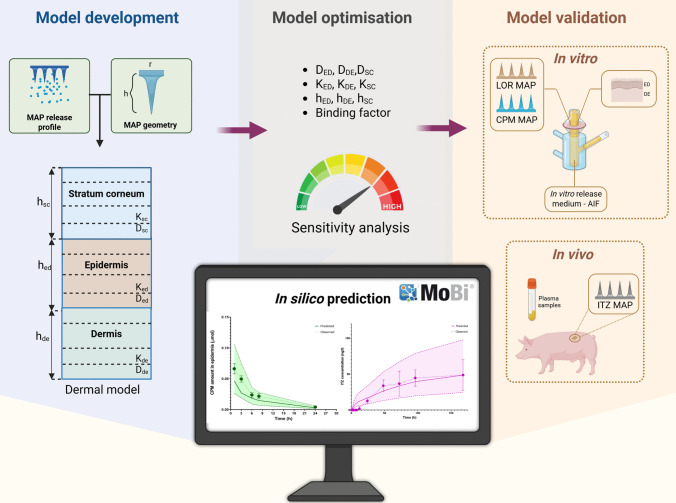

**Supplementary Information:**

The online version contains supplementary material available at 10.1007/s13346-025-01974-x.

## Introduction

The skin has long been recognised as a viable and effective route for drug delivery. To overcome its protective barrier, various delivery systems have been developed. Among these, dissolvable microarray patches (MAP) stand out for their unique ability to mechanically disrupt the stratum corneum (SC), enabling the release of drug cargo into deeper skin layers, thus facilitating both local and systemic treatments [1, 2]. Despite significant research and technological advancements, a pharmaceutical dissolvable MAP has yet to be commercialised. Among the several obstacles to their clinical translation are the absence of standardised preclinical assessment protocols and well-defined product quality attributes, which make it difficult to consistently produce systems that meet strict quality, safety, and efficacy standards [3].

Preclinical testing of dissolvable MAP involves various in vitro methods to evaluate drug release, its penetration through the skin layers, and permeability into simulated systemic circulation. However, these methods are often oversimplified, failing to accurately replicate the complex environment of in vivo skin [4]. Experimental variables, including skin metabolism, age, gender, and application site, significantly influence the results and their translatability to clinical settings [5]. These challenges extend to in vivo studies, where the choice of animal model and sampling methods can profoundly affect data interpretation [6]. Moreover, preclinical studies are often costly and associated with numerous ethical concerns [7].

Physiologically Based Pharmacokinetic (PBPK) modelling offers a solution to many of these challenges by enhancing the precision and reliability of both in vitro and in vivo studies. PBPK models integrate drug-specific properties with detailed physiological and biological knowledge to create a mechanistic framework that predicts drug behaviour in biological systems [8]. Recognised for their predictive accuracy, these models are increasingly endorsed by regulatory agencies for guiding decisions on drug-drug interactions, enzyme activity, pharmacogenetics, and therapy optimisation [9]. Efforts have been made to optimise PBPK models for various routes of administration, including dermal applications [10–12]. As a result, dermal models have been integrated into commercially available software programs such as GastroPlus^®^ (Simulations Plus, USA), Simcyp™ (Certara, UK), and MoBi^®^ (Open System Pharmacology). However, the application of PBPK modelling to the development of MAP products remains unexplored. Yadav et al. [13] attributed the limited progress in modelling and simulation tools for MAP to two main factors, a lack of sufficient parameters for relevant theoretical frameworks and limited expertise and interest in modelling these systems. Despite these challenges, the fundamental principles of drug pharmacokinetics through the skin are well-established [14–16], and experimental approaches can provide the necessary input parameters. Therefore, adapting existing dermal models for MAP is feasible and warrants investigation.

Existing dermal models primarily focus on conventional topical and transdermal systems, which are applied to the skin surface. However, MAP penetrate the skin and can deliver drugs into all three layers – stratum corneum (SC), viable epidermis (ED), and dermis (DE). The geometry of MAP can vary significantly depending on their design, critically influencing their skin penetration and drug distribution [17, 18]. Consequently, adapting PBPK models to account for these variations is essential. Moreover, MAP are fabricated using diverse methods, often incorporating polymeric matrices to control drug release rates [19]. The drug release rate from MAP determines the partitioning of the drug between the microneedle and the skin, influencing subsequent processes such as diffusion through skin layers, metabolism, and potential systemic absorption. Hence, incorporating drug release kinetics into modelling approaches is critical.

Elucidating the factors that influence drug movement across different skin layers can enhance the therapeutic efficacy of treatments for diseases such as skin cancer, psoriasis, and certain allergic or fungal conditions that require precise targeting of specific layers [20–23]. Therefore, simulation outcomes should ideally provide insights into drug kinetics within both the skin and systemic circulation. For drugs that undergo systemic exposure, PBPK models should also account for the redistribution of the active ingredient between the skin and systemic circulation. This is often achieved using whole-body-skin models to ensure accurate predictions [24]. These predictions rely on numerous input parameters related to a drug's physicochemical properties and skin-specific biological factors, which can vary significantly due to inter and intra-subject, as well as intra-study variability [24]. When experimental data are unavailable, quantitative structure–property relationships (QSPR) models or other computational methods can be used to estimate parameters; however, these approaches may introduce a degree of uncertainty. Sensitivity analysis and parameter optimisation are valuable tools for improving model reliability [24, 25]. However, it is important to select scientifically sound parameters, ensuring they are mechanistically relevant and represent data that cannot be obtained experimentally.

While the primary goal of PBPK models is to predict drug product in vivo behaviour, they can also play a crucial role in guiding experimental design during the early stages of product development. One commonly used test to evaluate drug distribution between the skin layers and release medium following MAP application is In Vitro Permeation Testing (IVPT). However, IVPT studies often suffer from high variability and low reproducibility [26]. Verified and validated PBPK models can serve as valuable tools for assessing the validity of IVPT results, informing further experimental studies and reducing both time and resources required for testing [12].

Building on the considerations outlined above, in this study we have developed a PBPK model designed to accurately predict drug distribution across skin layers and systemic absorption following MAP application. To achieve this, the dermal model from MoBi^®^ was specifically adapted for prediction of dissolvable MAP behaviour. This process involved incorporating microneedle geometry and in vitro release profiles for two types of MAP: a polymeric MAP loaded with the hydrophilic drug chlorpheniramine maleate (CPM), and drug-only MAP containing the lipophilic compounds loratadine (LOR) and itraconazole (ITZ). Uncertain input parameters related to skin thickness and drug-skin diffusion, partitioning and binding were carefully analysed and refined to improve the model's predictive accuracy. The optimised model was validated using experimental data from CPM and LOR MAP IVPT on porcine skin, as well as in vivo preclinical studies of ITZ MAP in a pig model, demonstrating robust performance across various drug molecules and experimental conditions.

## Materials and methods

### Materials

LOR (CAS 79794–75-5, purity > 98%), CPM (CAS 113–92-8, purity > 98%), and ITZ (CAS 84625–61-6, purity > 98%) were obtained from KEMPROTEC Limited (UK). Polyvinylpyrrolidone/vinyl acetate (PVP/VA; Kollidon^®^ VA 64, K-value 28, relative viscosity 1.178–1.55 for 1% aqueous solution) was supplied by BASF SE (Germany). Acetonitrile, methanol (both HPLC gradient-grade), and formic acid were sourced from Sigma-Aldrich (Ireland). Glacial acetic acid and HPLC-grade water were purchased from Fisher Scientific (UK). All other chemicals used were of pharmaceutical grade and were utilised as received.

### PBPK model development

#### MAP design

In this study, we used dissolvable MAP fabricated and characterised as previously described [27, 28]. LOR, CPM, and ITZ MAP comprised 25 pyramidal microneedles (5 × 5), each 500 μm in height and 333 μm in base width, with 2.2 mm base-to-base spacing and a total array area of approximately 1 cm^2^.Drug amount loaded in MAP was 0.5 ± 0.09, 0.1 ± 0.02 and 0.4 ± 0.09 mg per patch for LOR, CPM and ITZ MAP, respectively. The penetration depth of the dissolvable MAP was experimentally determined for all formulations using the Parafilm M^®^ insertion method [29], and was found to be approximately 70% of the total microneedle length (Fig. S1, Supplementary Information). Model development and optimisation were performed using porcine ear skin, with the thicknesses of the skin layers estimated to be 21 μm (SC), 72 μm (ED), and 1500 μm (DE), based on previous reports [30].

#### In vitro release testing (IVRT)

In vitro release testing (IVRT) was conducted to determine drug release rates for model development. IVRT was performed following established protocol [27]. MAP were immersed in the release medium and incubated at 37 °C under constant agitation. To better mimic physiological conditions, the biorelevant medium artificial interstitial fluid (AIF) was used, with its composition described previously [27, 31]. To ensure sink conditions, AIF was supplemented with 2% w/v polysorbate-80 for LOR MAP and 1% w/v sodium lauryl sulfate for ITZ MAP. IVRT was conducted for 24 h for LOR MAP, 1 h for CPM MAP, and 144 h for ITZ MAP, with sampling performed at designated time points. The varying durations of the release studies were chosen based on differences in release rates to ensure complete drug release. Drug concentrations were quantified using previously reported HPLC methods [27, 28]. The cumulative drug release data at each time point were incorporated into the dermal model in the MoBi^®^ software.

#### Development of dermal model for dissolvable MAP

**Fig. 1:**
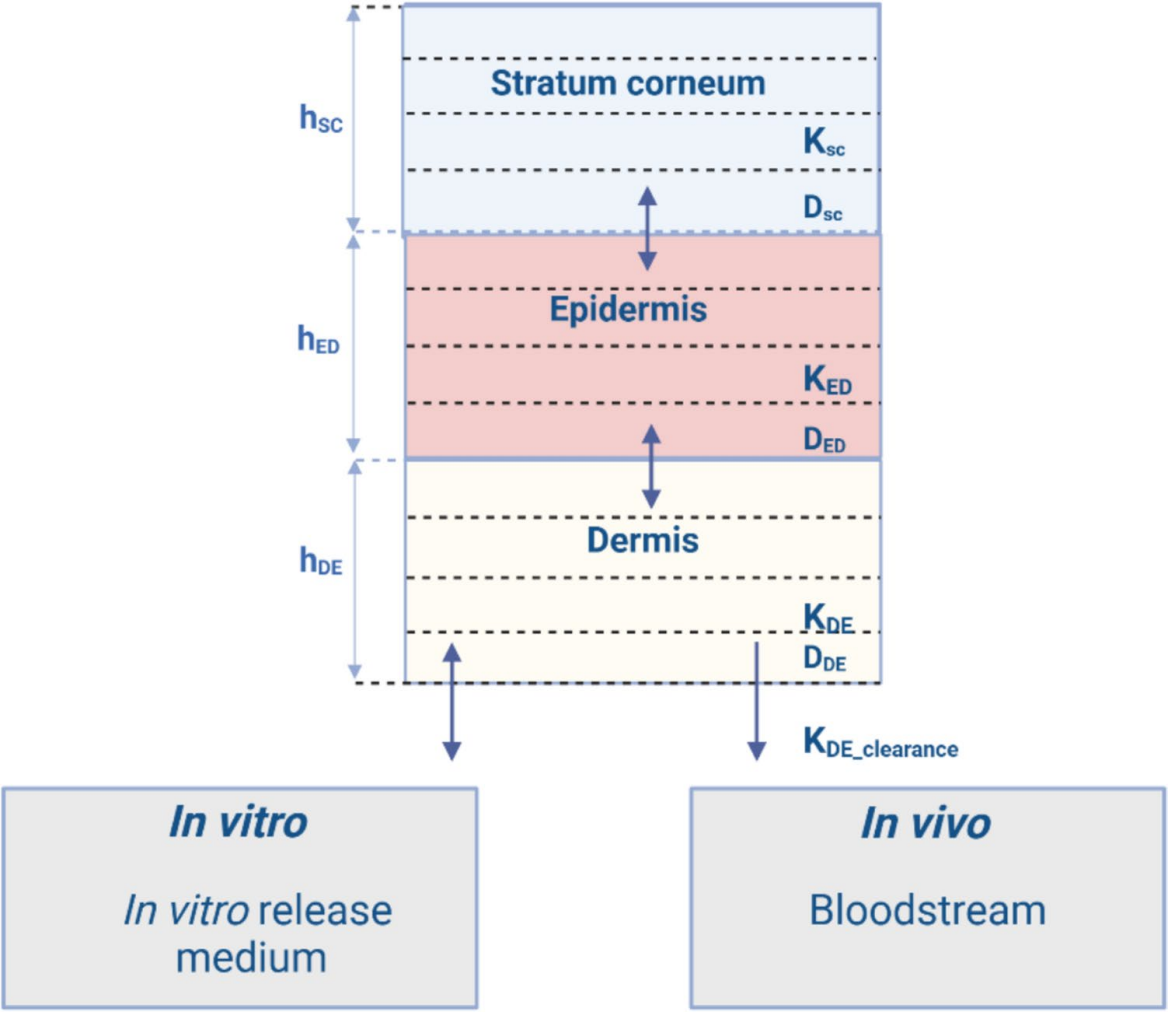
Schematic representation of the dermal model in MoBi^®^. Each skin layer is illustrated with four sublayers, which are further divided into ten sections (not shown in the illustration). Abbreviations: h_sc_, h_ED_ and h_DE​_ denote the thicknesses of the stratum corneum, epidermis, and dermis, respectively. K_sc_, K_ED_ and K_DE​_ represent the drug partition coefficients for the stratum corneum, epidermis, and dermis, while K_DE_clearance_​ corresponds to the clearance rate constant for drug removal from the dermis into systemic circulation. D_sc_, D_ED_ and D_DE_​ denote the diffusion coefficients of the drug in the respective skin layers. Created with BioRender.com

The dermal model in MoBi^®^ software (v.11) was developed based on the theoretical aspects of skin permeation described by Dancik et al. [16]. This model represents the skin as a multilayered slab consisting of three compartments which represent skin layers: SC, ED and DE (Fig. 1). Each layer inherits diffusion parameters based on its physical and chemical properties. The overall permeability in each skin layer is described by the diffusion coefficient (*D*_SC_, *D*_ED_, *D*_DE_) and partitioning coefficient (*K*_SC_, *K*_ED_, *K*_DE_). Within each layer, the drug can partition into four sublayers, which represent different phases, and each sublayer is further divided into ten smaller sections. Drug diffusion between these sections is governed by the ordinary differential equation [Eq. 1]:

1 $$\frac{d{A}_{SL,n}}{dt}={D}_{SL}*\left({C}_{SL, n-1}- {C}_{SL, n}\right)*\frac{{A}_{r}}{{dh}_{SL}}$$

where A_SL,n_ is the amount of compound in the n^th^ section of skin layer SL (i.e. SC, DE or ED), D_SL_ is the skin layer-specific diffusion coefficient, C_SL,n-1_ is the concentration of compound in the n-1th section of skin layer SL, C_SL,n_ is the concentration of compound in the n^th^ section of skin layer SL, Ar is the application surface area and dh_SL_ is the skin layer-specific thickness of one section.

Drug transition between the SC and ED layers, as well as between the ED and DE layers, is modeled as follows [Eq. 2] [Eq. 3]:

2 $$\frac{d{A}_{SC}}{dt}={A}_{r}*\left(\frac{\left(\frac{{D}_{SC}}{{h}_{SC}}\right)*(\frac{{D}_{ED}}{{h}_{ED}})}{\left(\frac{{D}_{SC}}{{h}_{SC}}\right)+\left(\frac{{K}_{ED/SC}*{D}_{ED }}{{K}_{SC/ED}*{h}_{ED}}\right)} \right)\left(\left(\frac{{K}_{ED/SC}*{C}_{SC}}{{K}_{SC/ED}}\right)-{C}_{ED}\right)$$

3 $$\frac{d{A}_{ED}}{dt}={A}_{r}*\left(\frac{\left(\frac{{D}_{ED}}{{h}_{ED}}\right)*(\frac{{D}_{DE}}{{h}_{DE}})}{\left(\frac{{D}_{ED}}{{h}_{ED}}\right)+\left(\frac{{K}_{DE/ED}*{D}_{DE }}{{K}_{ED/DE}*{h}_{DE}}\right)} \right)\left(\left(\frac{{K}_{DE/ED}*{C}_{ED}}{{K}_{ED/DE}}\right)-{C}_{DE}\right)$$

where A represents the amount of drug in the respective skin layer (SC or ED), D is the diffusion coefficient in the corresponding layer (SC, ED, or DE), K is the partition coefficient between the adjacent layers (SC/ED or ED/DE), h represents the thickness of the respective layer, c is the drug concentration, and Ar​ is the application surface area.

In DE, permeation is similarly modelled as one-dimensional diffusion for in vitro simulations [Eq. 4]:

4 $$\frac{{dA}_{DE}}{dt}={D}_{de}*\frac{{C}_{DE}}{dh}*{A}_{r}$$

where A_DE_​ is the amount of drug in the DE, D_DE_​ is the diffusion coefficient in the dermis, h is the layer thickness, C_DE_​ is the drug concentration in DE, and Ar is the application surface area.

For in vivo conditions, however, an additional clearance term is included to account for the drug's removal into the bloodstream [Eq. 5]:

5 $$\frac{d{A}_{DE}}{dt}={k}_{DE}*dh*Ar*{c}_{DE}$$

where, k_DE_ is dermal clearance rate.

**Fig. 2:**
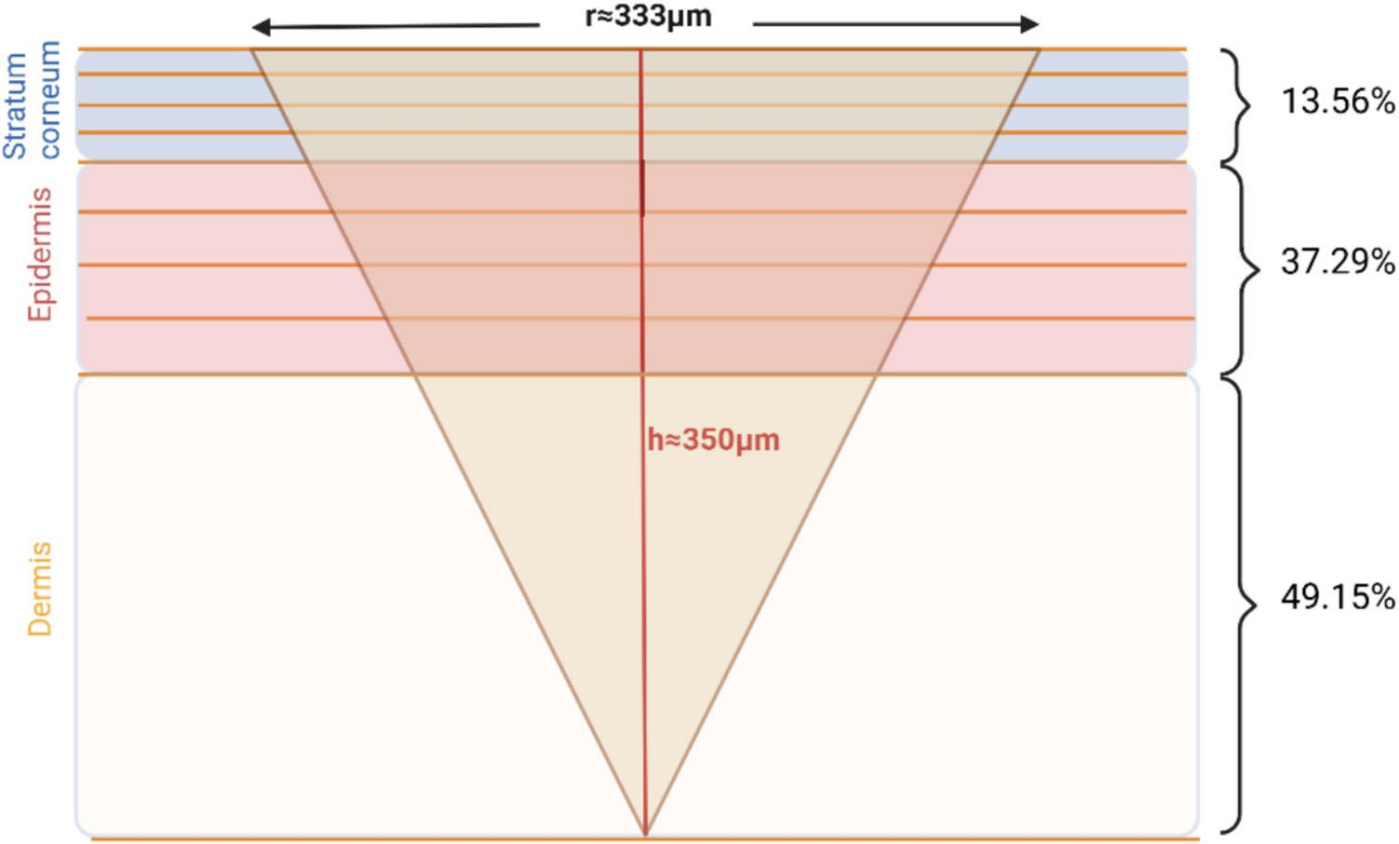
Distribution of drug across skin layers following MAP application. Each of the three skin layers is divided into four sublayers. After MAP application, the drug is distributed in the stratum corneum, epidermis, and the first layer of dermis. The percentages of the total drug loaded into the MAP are shown for each skin layer. Created with BioRender.com

Although the original dermal model in MoBi^®^ was designed for topical and transdermal formulations where the drug is applied on the SC surface, we adapted it for use with dissolvable MAP. Based on the MAP design and skin thickness, we determined that the microneedles would penetrate all sublayers of both SC and ED, as well as the first sublayer of DE. Using the microneedle geometry and the distribution of the drug across the layers, we then calculated the percentage of total drug amount present in each skin sublayer immediately following MAP application (Fig. 2). To implement this in MoBi^®^, we created nine separate applications, assigning the calculated drug amount to each corresponding skin sublayer. Since drug distribution within the smaller sections (10 sections within each sublayer) occurs rapidly, we did not model separate applications for these smaller compartments.

In addition to modelling drug distribution following microneedle application, it was essential to account for the drug release profile, as only the drug released from the MAP can penetrate through the skin layers and achieve the desired therapeutic effect. To achieve this, the model incorporated a time-dependent release fraction, which was directly implemented as a table of in vitro release data. At each time point, the available drug amount for permeation was determined as the product of the initial dose and the experimentally derived release fraction.

The original dermal model, developed for topical formulations, includes parameters for drug distribution into hair follicles and evaporation into the air. As these processes are not applicable to dissolvable MAP, where the drug is delivered directly into the skin, the corresponding parameters were set to zero in our simulations.

Taking into account considerations above, following assumptions were made:

1. Model assumes uniform insertion of microneedles across the skin. Variations in penetration depth or angle during duration of experiment could affect drug distribution;
2. Only drug released from MAP can transverse across skin layers and reach systemic circulation;
3. Presence of a polymer matrix in MAP formulation is not considered to influence drug diffusion through the skin;
4. Drug does not enter the hair follicles.

For the LOR and CPM MAP, an adopted dermal model was used for in vitro application. However, to track ITZ concentrations in plasma, a combined whole body- dermal model was employed, as described below.

#### Whole body model for ITZ

The simulation of ITZ plasma concentrations following oral administration in pigs was performed using the PK-Sim software. The minipig model, developed and implemented in PK-Sim as outlined by Suenderhauf and Parrott [32], was adapted to our study. The input parameters used for the simulation are detailed in Table 1. The accuracy of the simulation results was confirmed by comparison with previously published in vivo data [28]. Finally, the whole body model following oral administration of ITZ was integrated with the dermal model using the MoBi^®^ software.

**Table 1:** Input parameters for the ITZ whole body model

Input parameter	Value
Molecular weight (g/mol)	705.64 [34]
LogP	6.5 [35]
Fraction unbound (%)	0.02 [34]
pKa	3.7 [34]
Solubility at pH7 (g/l)	0.0096 [35]
Solubility gain per charge	1000 [36]
Intrinsic clearance (l/min)	278 [37]
Dose (mg)	50 [28]
Calculation method for Partition coefficients and Cellular Permeabilities	PK-Sim Standard

The dissolution profile of ITZ capsules in biorelevant medium was obtained from Thiry et al. [33]

### PBPK model optimisation

The input parameters for the dermal model, including the drugs’ physicochemical properties, are provided in Table 2. Other critical parameters influencing drug diffusion (*D*_SC_, *D*_ED_, *D*_DE_), partitioning (*K*_SC_, *K*_ED_, *K*_DE_) and binding (Binding factor), as well as biological parameters such as skin thickness, underwent sensitivity analysis if they were unavailable, unreliable, or not directly derived from experimental data or literature. Supplementary Table S1 provides a detailed overview of these parameters, including their descriptions and equations. Sensitivity analysis was performed to assess their impact on model outcomes, and highly sensitive parameters were further optimised using the Levenberg–Marquardt algorithm.

**Table 2:** Physicochemical input parameters for selected drug molecules used in the PBPK model

Input parameter	LOR	CPM	ITZ
C atoms	22	20	35
Cl atoms	1	1	2
Compound type	Base	Base	Base
Double bonds	7	9	13
Experimental denstity (g/cm^3^)	1.30 [38]	1.28 [39]	1.27 [40]
Experimental water solubility (g/cm3)	1.34X10^−5^ [35]	0.05 [35]	9.64X10^−6^ [35]
Fraction unbound (%)	3 [41]	30 [42]	0.02 [34]
H atoms	23	23	35
SC hydration	hydrated	hydrated	hydrated
Skin property	In vitro	In vitro	In vivo
logP	4.80 [35]	3.62 [35]	6.5 [35]
Melting temperature (°C)	135 [35]	133 [35]	168 [35]
Molecular weight (g/mol)	382.88 [41]	390.9 [43]	705.60 [34]
N atoms	2	2	8
O atoms	2	4	2
Rings	4	2	4
Skin surface temperature (°C)	32	32	32
Strongest base pKa	4.5 [41]	9.47 [43]	[34]

### PBPK model validation

#### In vitro validation

The model was validated for the in vitro performance of LOR and CPM MAP using data obtained in IVPT studies. These studies were conducted in vertical Franz diffusion cells (PermeGear, USA) with porcine ear skin as the membrane and biorelevant AIF as the receptor medium. MAP were inserted into the prepared skin samples using an in-house applicator (25 N) and secured in the donor compartment with adhesive tape, following a protocol described in detail previously [27], with modifications to the sampling procedure. In addition to collecting samples from the release medium, skin samples were removed from the setup, and the ED and DE layers were separated using a heat separation method [44] at each time point (1, 3, 6, 8 and 24 h). Drug content in each layer was then quantified using previously described and validated HPLC methodology [27, 28]. The experimental data for drug amounts in the ED, DE, and in the in vitro release medium (referred as ‘in vitro sink’ in MoBi^®^) were compared with model predictions. Absolute average fold error (AAFE) between predicted and observed curves was calculated using a previously reported equation [45]. Models with AAFE values between 1 and 2 were deemed successfully verified. Additionally, observed and predicted data were plotted to visually assess whether the predicted values fell within a two-fold range of the observed values.

#### In vivo validation

The model was validated for in vivo performance using data from ITZ MAP in vivo studies conducted on Landrace pigs, as previously described by Sartawi et al. [28]. These studies utilized MAP with the same geometry as noted above, but with a larger array (14 × 14), corresponding to a total ITZ dose of 4.1 mg. The PBPK model was verified if the AAFE for concentration–time profiles and pharmacokinetic parameters (C_max_, T_max_, AUC_0→24 h_, and AUC_0→168 h_) was between 1 and 2, and if the ratio of predicted to observed pharmacokinetic parameters fell within 0.5 to 2.

## Results

### PBPK model development and optimisation

**Fig. 3:**
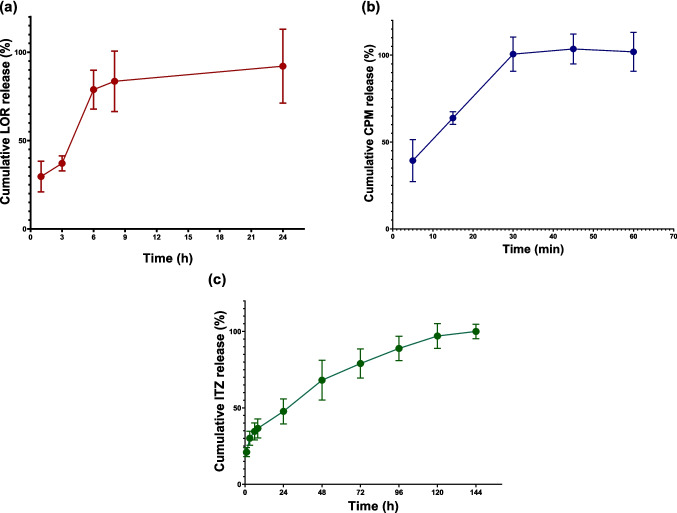
In vitro drug release profile from MAP in biorelevant media: (**a**) CPM; (**b**) LOR and (**c**) ITZ (*n* = 3.). Drug release profile significantly varied between the proposed formulations. CPM exhibited rapid release, while LOR and ITZ demonstrated prolonged release. Y error bars indicate the standard deviation

The PBPK model for dissolvable MAP was developed by integrating their geometry and the drug release profiles into an existing dermal model in MoBi^®^. The geometry was used to estimate the percentage of the total dose distributed in skin layers and sublayers. The drug release profiles in the biorelevant media determined through IVRT studies are presented in Fig. 3. CPM exhibited a rapid release profile, with an initial burst of 39.3 ± 12.1% of drug released within the first 5 min, followed by complete drug release within 30 min. In contrast, LOR and ITZ displayed prolonged release profiles, with complete drug release occurring after 24 and 120 h, respectively. The fraction of drug released at each time point predetermined the amount of drug dose available for permeation in the skin layers.

**Fig. 4:**
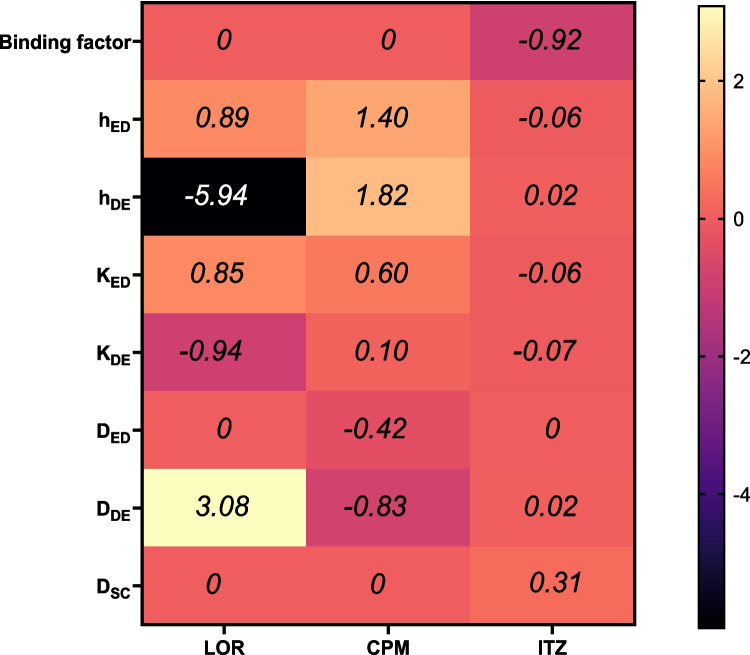
Heatmap showing a sensitivity analysis of the most influential parameters for model predictions across three drug formulations: LOR, CPM, and ITZ MAP. Sensitivity coefficients farther from 0 indicate a stronger influence on model outcomes. Positive values correspond to an increase, and negative values indicate a decrease in the model outcome, reflecting the direction of each parameter's effect. For LOR MAP, key parameters include dermal thickness, dermal diffusion coefficient, dermal partition coefficient, epidermal thickness, and epidermal partition coefficient. For CPM MAP, dermal thickness, dermal diffusion coefficient, and epidermal thickness were the most significant factors. The binding factor was identified as the most influential parameter for ITZ MAP. Abbreviations: h_ED_, h_DE_- thickness of epidermis and dermis; K_ED_, K_DE_ – partition coefficients in epidermis and dermis; D_ED_, D_DE_, D_SC,_ – diffusion coefficients in epidermis, dermis and stratum corneum

To refine the PBPK model parameters and enhance predictive accuracy, a sensitivity analysis was performed to identify the parameters with the greatest influence on model outcomes for each drug. For LOR MAP, the most critical parameters influencing predictions were identified as dermal thickness, dermal diffusion and partition coefficients, as well as epidermal thickness and epidermal partition coefficient. Similarly, for CPM MAP, dermal thickness, the dermal diffusion coefficient, and epidermal thickness were the key determinants of model outcomes. In contrast, for ITZ MAP, the binding factor emerged as the most influential parameter affecting plasma drug concentration predictions (Fig. 4). These findings guided subsequent parameter optimisation to improve the model's fit and reliability. Optimised parameter values are presented in Table 3, and figures showing predicted vs observed data prior to optimisation can be found in the Supplementary Information (Fig. S2-S4).

**Table 3:** Key parameters identified in sensitivity analysis for LOR, CPM, and ITZ MAP: starting and optimal values

Drug	Parameter	Starting value	Optimal value
**LOR**	D_DE_ (cm^2^/s)	3.32 × 10^–8^	5.34 × 10^–8^
	K_DE_	44.67	38.55
	K_ED_	44.67	46.00
	Dermal thickness (µm)	1500	1439.42
	Epidermal thickness (µm)	72	85
**CPM**	D_DE_ (cm^2^/s)	2.52 × 10^–7^	2.79 × 10^–7^
	Dermal thickness (µm)	1500	1237.02
	Epidermal thickness (µm)	72	80
**ITZ**	Binding factor	2101.87	1262.99

D_DE_ diffusion coefficient in dermis, K_DE_ partition coefficient in dermis, K_ED_ partition coefficient in epidermis

### PBPK model validation

#### In vitro model validation

The developed PBPK model was validated using in vitro performance data obtained from IVPT studies conducted with LOR and CPM MAP. Validation involved comparing the observed and predicted drug amounts in the ED, DE and the in vitro release medium.

**Fig. 5:**
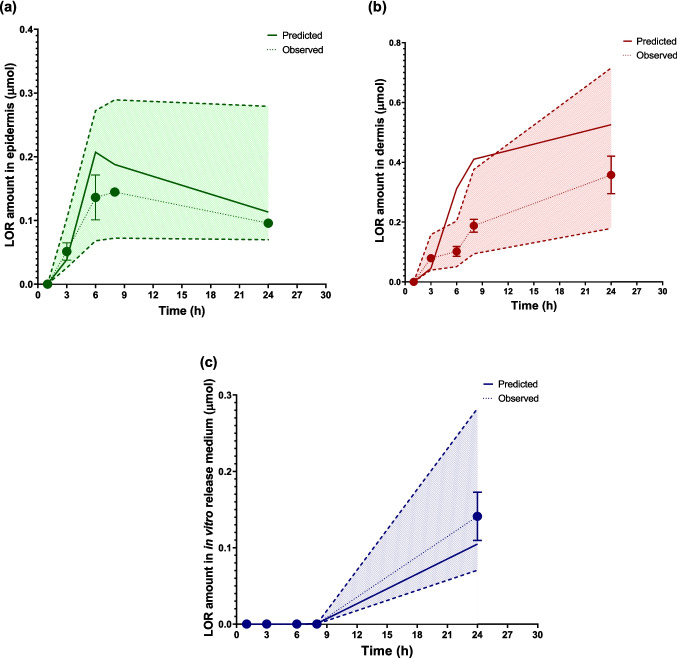
LOR distribution in skin layers and release medium over time: (**a**) epidermis, (**b**) dermis, and (**c**) cumulative release in in vitro release medium (*n* = 2). Solid lines represent model predictions, dots represent in vitro observed data, and the shaded region indicates the two-fold error margin for predicted values. For the dermis, the predicted amounts at the 6- and 8-h time points were overestimated. Y error bars indicate the standard deviation

The AAFE values, calculated between the predicted and observed curves, were 1.42 for the ED, 1.53 for the DE, and 1.37 for the in vitro release medium. As shown in Fig. 5a–c, the predicted amounts of LOR generally fell within a two-fold range of the observed data. A discrepancy was observed in DE, where the model overpredicted drug concentrations at the 6- and 8-h time points compared to the observed values. Although concentrations at these two timepoints fell outside the two-fold range of the observed data, the AAFE of 1.53 indicates that the model's overall accuracy remains within an acceptable error margin.

**Fig. 6:**
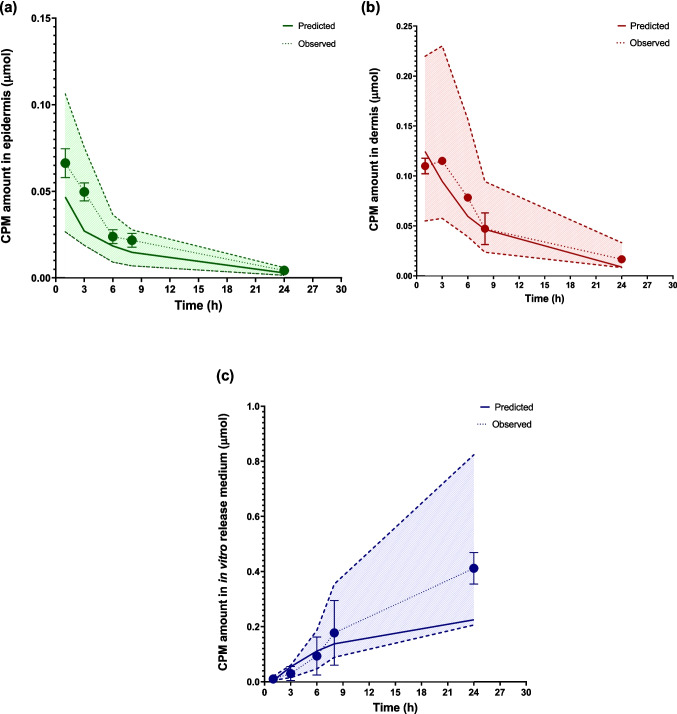
CPM distribution in skin layers and release medium over time: (**a**) epidermis, (**b**) dermis, and (**c**) cumulative release in in vitro release medium (*n* = 2). Solid lines represent model predictions, dots represent in vitro observed data, and the shaded region indicates the two-fold error margin for predicted values. All predicted data fall within this range. Y error bars indicate the standard deviation

Similarly, for CPM MAP, the calculated AAFE values for the predicted versus observed curves were 1.28 for the ED, 1.33 for the DE, and 1.37 for the in vitro release medium, reflecting good model accuracy. Additionally, the simulated CPM amounts consistently stayed within a two-fold range of the observed data across the ED, DE, and in vitro release medium, as shown in Fig. 6a–c.

#### In vivo model validation

**Fig. 7:**
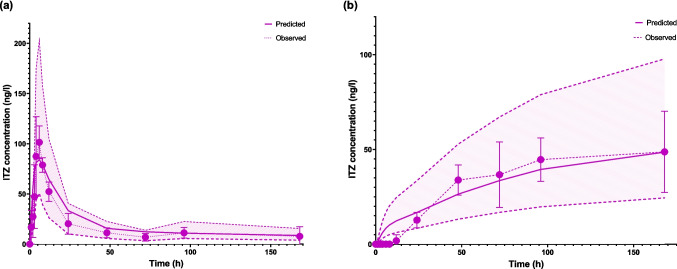
ITZ plasma concentrations after (**a**) oral administration of capsules and (**b**) MAP administration (*n* = 5). Solid lines represent model predictions, dots represent in vivo observed data, and the shaded region indicates the two-fold error margin for predicted values. For MAP, the predicted concentrations during the initial phase (0–24 h) were overestimated. Y error bars indicate the standard deviation

The whole-body model was validated using in vivo data for ITZ following oral administration in pigs. As shown in Fig. 7a, a strong correlation was observed between the measured and predicted values. Building on this validation, the model was integrated with a dermal model to simulate ITZ plasma concentrations following MAP application. This integrated model was further validated using in vivo data from pigs treated with ITZ MAP. The AAFE was calculated for the concentration–time profile from 12 to 168 h, as the observed data at earlier time points (0–12 h) were zero, making it impossible to compute AAFE using the equation. The AAFE for the predicted versus observed profile during this period was 1.02, indicating a good fit. This was further supported by the observed versus predicted plot (Fig. 7b). Additionally, comparisons of pharmacokinetic parameters revealed that the AAFE for AUC_0→168 h​,_ C_max_, and T_max_ values fell within the proposed range of 1–2, while the ratio of predicted to observed values for these parameters was within the acceptable 0.5–2 range (Table 4). However, the ratio of predicted to observed AUC_0→24 h_ exceeded this range.

**Table 4:** Pharmacokinetic parameters calculated from observed and predicted data for MAP application on landrace pigs in vivo

	Observed	Predicted	AAFE	Ratio
AUC_0→24 h_ (ng hr^−1^ ml)	90.9 ± 53.1	263.8	2.90	2.90
AUC_0→168 h_ (ng hr^−1^ ml)	5233.7 ± 2669.1	5546.5	1.06	1.06
C_max_ (ng ml^−1^)	52.5 ± 20.5	49.1	1.07	0.94
t_max_ (h)	144 ± 41.6	155.3	1.08	1.08
Profile _12-168h_	-	-	1.02	-

Observed data are presented as mean ± standard deviation (*n* = 5). Abbreviations: *AAFE* absolute average fold error, AUC0 → 24 h—area under the curve from 0 to 24 h; AUC0 → 168 h—area under the curve from 0 to 168 h, Cmax—maximum concentration of the drug in plasma, tmax—time at which Cmax occurs

## Discussion

The application of PBPK modelling in MAP development is valuable as it can enhance preclinical and clinical studies, address associated ethical concerns by reducing reliance on animal testing and enabling more informed, rational design of clinical studies—ultimately accelerating the market introduction of these products. However, further research is needed to develop models that can accurately capture the complex interactions between MAP design and the skin environment. In this study, we investigated the optimisation of a dermal model available in the MoBi^®^ software to predict drug kinetics following MAP application.

The first optimisation step involved adjusting the existing dermal model to accommodate the geometry of MAP. By considering the geometric properties and the drug loading per patch, it is possible to calculate the percentage of the total dose delivered to each skin layer, even for varying geometries. Nevertheless, several limitations related to MAP design should be considered. Microneedle penetration depth has been identified as the most critical determinant of effective skin permeability [46] and insertion has been shown to depend on factors such as needle geometry, formulation composition, and application force [47, 48]. Therefore, each formulation should be experimentally tested for insertion depth prior to inputting data into the model, ideally using real-time methodologies [48] that can provide more accurate measurements than the Parafilm^®^M method we used for our estimations, thereby enhancing predictive accuracy. Another limitation concerns scaling to higher doses and larger patches*. *In vivo validation with a 14 × 14 MAP demonstrated that the model can simulate higher drug loads, with drug amounts proportionally distributed across skin layers; however, it does not explicitly capture potential effects of increased microneedle density, such as the “bed of nails” phenomenon [49], which may influence delivery efficiency. The model also assumes a uniform MAP design without a backing layer, and future refinements should consider potential back-migration of the drug to the baseplate, particularly for hydrophilic compounds [50], as this could affect prediction accuracy. Finally, while the model incorporates experimentally derived drug release profiles to capture formulation-specific dissolution, it does not explicitly include particle size as an input parameter, limiting its ability to mechanistically predict the influence of particle size on overall pharmacokinetic behaviour—an aspect increasingly recognised as important in the MAP field [51].

In addition to adapting the model to MAP geometry, the release behaviour of drugs from MAP is essential for determining the amount of drug available for partitioning and diffusion through the skin layers. IVRT in biorelevant media is a straightforward method for gaining insight into a drug's release profile from MAP. While AIF is designed to closely mimic physiological conditions [27], challenges arise in quantifying lipophilic compounds and maintaining sink conditions. To address this, surfactants were added to the medium composition. However, these solubility enhancers can alter the release profile, potentially leading to inaccurate insights into drug release behaviour and, consequently, inaccurate PBPK predictions. Our findings indicate that predictions were more accurate for MAP containing CPM, a hydrophilic drug, compared to LOR and ITZ, both highly lipophilic. This underscores the need for biorelevant media to enhance understanding of drug release behaviour in vivo and improve PBPK model accuracy. It also highlights the importance of further research to adapt such media for drugs with varying physicochemical properties.

The confidence in PBPK models increases when input parameters are tailored to the specific drug and experimental design. However, due to the complexity of the equations used to build these models, it is not possible to obtain all necessary parameters. Moreover, experimentally derived values may not always reflect in vivo conditions. In this context, sensitivity analysis and parameter optimisation become essential. However, parameter selection should not be arbitrary—it must be based on a deep understanding of the parameters and their influence on model outcomes. In dermal models, diffusion and partition coefficients across various skin layers are crucial for predicting drug kinetics, but are often difficult to determine experimentally, with high uncertainty and variability [24]. For both LOR and CPM MAP, these parameters exhibited high sensitivity, and their optimisation significantly improved the fit with observed data. Another sensitive parameter is the thickness of the epidermal and dermal layers. Input values were derived from prior studies measuring porcine skin thicknesses, though it is important to recognise that these are average values, with inherent variability even within the same experimental setup. In addition, another study reported slightly different values for pig ear thickness [52]. As it is not practically feasible to measure skin thickness prior to each experiment, we demonstrated that adjusting this parameter within a physiologically relevant range can further improve the predictive accuracy of the model. The binding factor, which accounts for drug binding to skin proteins, was identified as a particularly sensitive parameter for ITZ. This is unsurprising given ITZ’s high affinity for skin tissue, which allows it to be detectable in the skin even weeks after treatment [53].

By optimising these parameters, our model successfully predicted the distribution of CPM and LOR in different skin layers in IVPT studies. Given the small drug quantities involved and the high sensitivity and variability of IVPT [26], the PBPK model served as an effective tool for verifying our IVPT experimental design, offering significant value in early preclinical studies. AAFE was employed as the primary metric to evaluate the goodness-of-fit and to address potential biases inherent in the two-fold error range method [54]. Although the predicted curve for LOR did not fall within the two-fold error range of the observed values at the 6- and 8-h time points in DE, the AAFE value of 1.53 indicated that the overall model validation was acceptable. Beyond the error fold values, Tsakalozou et al. emphasized the importance of evaluating the overall shape of the curve for model validation [24], which further reassured that the model still accurately captures LOR pharmacokinetics. For CPM, the correlation between observed and predicted data was strong. The predicted curve closely mirrored the shape of the observed data and fell within the two-fold error range, indicating a high level of reliability. While we validated the model's ability to predict IVPT outcomes for MAP with drugs of different physicochemical properties, applying the proposed model to different skin models—such as those from different species, anatomical regions, or varying thicknesses—could further assess its predictive accuracy and enhance its utility in IVPT.

The optimised dermal model was also validated against in vivo data from ITZ MAP studies conducted on Landrace pigs. The model overpredicted plasma drug concentrations during the first 24 h. This discrepancy may be due to two factors. First, the presence of sodium lauryl sulfate in the in vitro release medium, which was required to maintain sink conditions for ITZ, likely accelerated drug release compared to in vivo conditions. Although the use of biorelevant media is recommended for more accurate predictions [8], challenges remain for very poorly soluble drugs like ITZ. Second, due to ITZ's strong affinity for skin proteins and its slower release at early time points, it is possible that plasma drug concentrations were below the quantification limit. Despite this, there was a strong alignment between predicted and observed data from 24 to 168 h, both in terms of the curve shape and the desired ratio between the values. This suggests that the model holds promise for predicting in vivo pharmacokinetics of MAP.

One of the key advantages of the proposed model is its ability to quantify drug concentrations in both skin layers and plasma, a capability not reported in previous models for dissolvable MAP [45, 55, 56]. However, due to the lack of observed data from different skin layers, we were unable to validate this aspect of the model. While in vivo preclinical studies often focus on measuring drug concentrations in plasma, shifting the focus to tracking drug distribution within skin layers could provide valuable insights into drug penetration following MAP application and enhance the validation of PBPK models.

## Conclusion

The development of PBPK models suitable for MAP is a critical step in advancing preclinical and clinical stages of product development while facilitating market entry. In this study, we successfully optimised the dermal model in the MoBi^®^ software by incorporating MAP geometry and drug release behaviour. The model validation using in vitro and in vivo data from three MAP formulations with varying physicochemical properties demonstrated its robustness across diverse experimental designs. Additionally, we highlighted the importance of mechanistic insights into key model parameters and their impact on outcomes. Further studies incorporating in vivo validation of drug kinetics in the skin could further enhance the model’s predictive accuracy. With the growing accessibility of PBPK software programs capable of simulating drug kinetics for various delivery systems, we anticipate that these findings can contribute to the seamless integration of MAP into such frameworks, enabling their simulation with specific input parameters and making them accessible to non-experts in modelling.

## Supplementary Information

Below is the link to the electronic supplementary material.

Supplementary file1 (DOCX 484 KB)

## Data Availability

The datasets generated during and/or analysed during the current study are available from the corresponding author on reasonable request.

## References

[1] Lahiji SF, Dangol M, Jung H. A patchless dissolving microneedle delivery system enabling rapid and efficient transdermal drug delivery. Sci Rep. 2015;5(1):7914. 10.1038/srep07914. 25604728 10.1038/srep07914 PMC4300505

[2] Paredes AJ, McKenna PE, Ramöller IK, Naser YA, Volpe-Zanutto F, Li M, et al. Microarray patches: poking a hole in the challenges faced when delivering poorly soluble drugs. Adv Funct Mater. 2021;31(1):2005792. 10.1002/adfm.202005792.

[3] Lutton REM, Moore J, Larrañeta E, Ligett S, Woolfson AD, Donnelly RF. Microneedle characterisation: the need for universal acceptance criteria and GMP specifications when moving towards commercialisation. Drug Deliv Transl Res. 2015;5:313–31. 10.1007/s13346-015-0237-z. 26022578 10.1007/s13346-015-0237-z

[4] Tsai PC, Lee S, Michniak-Kohn B. In vitro methods for screening transdermal formulations. Ther Deliv. 2015;6(9):1043–52. 10.4155/tde.15.58. 26419344 10.4155/tde.15.58

[5] Zsikó S, Csányi E, Kovács A, Budai-Szűcs M, Gácsi A, Berkó S. Methods to evaluate skin penetration in vitro. Sci Pharm. 2019;87(3):19. 10.3390/scipharm87030019.

[6] Railic M, Vucen S, Crean A. Insights into preclinical evaluation of dissolvable microarray patches. Int J Pharm. 2025;125361. 10.1016/j.ijpharm.2025.125361. 10.1016/j.ijpharm.2025.125361 39971167

[7] Zhuang X, Lu C. PBPK modeling and simulation in drug research and development. Acta Pharm Sin B. 2016;6(5):430–40. 10.1016/j.apsb.2016.04.004. 27909650 10.1016/j.apsb.2016.04.004 PMC5125732

[8] Kuepfer L, Niederalt C, Wendl T, Schlender JF, Willmann S, Lippert J, et al. Applied concepts in PBPK modeling: how to build a PBPK/PD model. CPT Pharmacometrics Syst Pharmacol. 2016;5(10):516–31. 10.1002/psp4.12134. 27653238 10.1002/psp4.12134 PMC5080648

[9] Lin W, Chen Y, Unadkat JD, Zhang X, Wu D, Heimbach T. Applications, challenges, and outlook for PBPK modeling and simulation: a regulatory, industrial and academic perspective. Pharm Res. 2022;39(8):1701–31. 10.1007/s11095-022-03274-2. 35552967 10.1007/s11095-022-03274-2

[10] van Osdol WW, Novakovic J, Le Merdy M, Tsakalozou E, Ghosh P, Spires J, et al. Predicting human dermal drug concentrations using PBPK modeling and simulation: clobetasol propionate case study. AAPS PharmSciTech. 2024;25(3):39. 10.1208/s12249-024-02740-x. 38366149 10.1208/s12249-024-02740-x

[11] Yun YE, Calderon-Nieva D, Hamadeh A, Edginton AN. Development and evaluation of an in silico dermal absorption model relevant for children. Pharmaceutics. 2022;14(1):172. 10.3390/pharmaceutics14010172. 35057066 10.3390/pharmaceutics14010172 PMC8780349

[12] Krumpholz L, Polak S, Wiśniowska B. Physiologically-based pharmacokinetic model of in vitro porcine ear skin permeation for drug delivery research. J Appl Toxicol. 2024;44(12):1936–48. 10.1002/jat.4687. 39134399 10.1002/jat.4687

[13] Yadav PR, Han T, Olatunji O, Pattanayek SK, Das DB. Mathematical modelling, simulation and optimisation of microneedles for transdermal drug delivery: trends and progress. Pharmaceutics. 2020;12(8):693. 10.3390/pharmaceutics12080693. 32707878 10.3390/pharmaceutics12080693 PMC7464833

[14] Potts RO, Guy RH. Predicting skin permeability. Pharm Res. 1992;9:663–9. 10.1023/A:1015810312465. 1608900 10.1023/a:1015810312465

[15] Mitragotri S. A theoretical analysis of permeation of small hydrophobic solutes across the stratum corneum based on scaled particle theory. J Pharm Sci. 2002;91(3):744–52. 10.1002/jps.10048. 11920759 10.1002/jps.10048

[16] Dancik Y, Miller MA, Jaworska J, Kasting GB. Design and performance of a spreadsheet-based model for estimating bioavailability of chemicals from dermal exposure. Adv Drug Deliv Rev. 2013;65(2):221–36. 10.1016/j.addr.2012.01.006. 22285584 10.1016/j.addr.2012.01.006

[17] Gittard SD, Chen B, Xu H, Ovsianikov A, Chichkov BN, Monteiro-Riviere NA, et al. The effects of geometry on skin penetration and failure of polymer microneedles. J Adhes Sci Technol. 2013;27:227–43. 10.1080/01694243.2012.705101. 23543070 10.1080/01694243.2012.705101 PMC3610923

[18] Min HS, Kim Y, Nam J, Ahn H, Kim M, Kang G, et al. Shape of dissolving microneedles determines skin penetration ability and efficacy of drug delivery. Biomater Adv. 2023;145:213248. 10.1016/j.bioadv.2022.213248. 36610239 10.1016/j.bioadv.2022.213248

[19] Singh P, Carrier A, Chen Y, Lin S, Wang J, Cui S, et al. Polymeric microneedles for controlled transdermal drug delivery. J Control Release. 2019;315:97–113. 10.1016/j.jconrel.2019.10.022. 31644938 10.1016/j.jconrel.2019.10.022

[20] Zhi D, Yang T, Zhang T, Yang M, Zhang S, Donnelly RF. Microneedles for gene and drug delivery in skin cancer therapy. J Control Release. 2021;335:158–77. 10.1016/j.jconrel.2021.05.009. 33984344 10.1016/j.jconrel.2021.05.009

[21] Raychaudhuri SK, Maverakis E, Raychaudhuri SP. Diagnosis and classification of psoriasis. Autoimmun Rev. 2014;13:490–5. 10.1016/j.autrev.2014.01.008. 24434359 10.1016/j.autrev.2014.01.008

[22] Pople PV, Singh KK. Targeting tacrolimus to deeper layers of skin with improved safety for treatment of atopic dermatitis. Int J Pharm. 2010;398:165–78. 10.1016/j.ijpharm.2010.07.008. 20637847 10.1016/j.ijpharm.2010.07.008

[23] Hay RJ. Fungal infections. Clin Dermatol. 2006;24:201–12. 16714201 10.1016/j.clindermatol.2005.11.011

[24] Tsakalozou E, Alam K, Babiskin A, Zhao L. Physiologically-based pharmacokinetic modeling to support determination of bioequivalence for dermatological drug products: scientific and regulatory considerations. Clin Pharmacol. 2022;111(5):1036–49. 10.1002/cpt.2356. 10.1002/cpt.2356 34231211

[25] Liu D, Li L, Rostami-Hodjegan A, Bois FY, Jamei M. Considerations and caveats when applying global sensitivity analysis methods to physiologically based pharmacokinetic models. AAPS J. 2020;22:1–13. 10.1208/s12248-020-00480-x. 10.1208/s12248-020-00480-x PMC7367914 32681207

[26] Oh L, Yi S, Zhang D, Shin SH, Bashaw E. In vitro skin permeation methodology for over-the-counter topical dermatologic products. Ther Innov Regul Sci. 2020;54:693–700. 10.1007/s43441-019-00104-3. 33301148 10.1007/s43441-019-00104-3

[27] Railic M, Crean AM, Vucen S. Unravelling microarray patch performance: the role of in vitro release medium and biorelevant testing. Mol Pharm. 2024;21(10):5028–40. 10.1021/acs.molpharmaceut.4c00459. 39195905 10.1021/acs.molpharmaceut.4c00459 PMC11462508

[28] Sartawi Z, Blackshields C, Ariamanesh A, Farag FF, Griffin B, Crean A, et al. Glass microneedles: a case study for regulatory approval using a quality by design approach. Adv Mater. 2023;35(52):2305834. 10.1002/adma.202305834. 10.1002/adma.202305834 37950607

[29] Larrañeta E, Moore J, Vicente-Pérez EM, González-Vázquez P, Lutton R, Woolfson AD, et al. A proposed model membrane and test method for microneedle insertion studies. Int J Pharm. 2014;472:65–73. 10.1016/j.ijpharm.2014.05.042. 24877757 10.1016/j.ijpharm.2014.05.042 PMC4111867

[30] Jacobi U, Kaiser M, Toll R, Mangelsdorf S, Audring H, Otberg N, et al. Porcine ear skin: an in vitro model for human skin. Skin Res Technol. 2007;13:19–24. 10.1111/j.1600-0846.2006.00179.x. 17250528 10.1111/j.1600-0846.2006.00179.x

[31] Bretag AH. Synthetic interstial fluid for isolated mammalian tissue. Life Sci. 1969;8(5):319–29. 10.1016/0024-3205(69)90283-5. 5781321 10.1016/0024-3205(69)90283-5

[32] Suenderhauf C, Parrott N. A physiologically based pharmacokinetic model of the minipig: data compilation and model implementation. Pharm Res. 2013;30(1):1–15. 10.1007/s11095-012-0911-5. 23179779 10.1007/s11095-012-0911-5

[33] Thiry J, Broze G, Pestieau A, Tatton AS, Baumans F, Damblon C, et al. Investigation of a suitable in vitro dissolution test for itraconazole-based solid dispersions. Eur J Pharm Sci. 2016;85:94–105. 10.1016/j.ejps.2016.02.002. 26850682 10.1016/j.ejps.2016.02.002

[34] Al-Badr AA, El-Subbagh HI. Itraconazole: comprehensive profile. Prof Drug Subst Excip Relat Methodol. 2009;34:193–264. 10.1016/S1871-5125(09)34005-4. 10.1016/S1871-5125(09)34005-4 22469175

[35] Wishart DS, Guo AC, Oler E, Wang F, Anjum A, Peters H, et al. HMDB 5.0: The human metabolome database for 2022. Nucleic Acids Res. 2022;50:622–31. 10.1093/nar/gkab1062. 10.1093/nar/gkab1062 PMC8728138 34986597

[36] Vasilev NA, Surov AO, Voronin AP, Drozd KV, Perlovich GL. Novel cocrystals of itraconazole: insights from phase diagrams, formation thermodynamics and solubility. Int J Pharm. 2021;590:120441. 10.1016/j.ijpharm.2021.120441. 10.1016/j.ijpharm.2021.120441 33675927

[37] Knox C WMKC et al. DrugBank 6.0: the drugbank knowledgebase for 2024. Nucleic Acids Res. 2024. 10.1093/nar/gkad976 10.1093/nar/gkad976 PMC10767804 37953279

[38] Shi Z, Wang C, Sun CC. Molecular origin of the distinct tabletability of loratadine and desloratadine: role of the bonding area – bonding strength interplay. Pharm Res. 2020;37:1–10. 10.1007/s11095-020-02856-2. 10.1007/s11095-020-02856-2 32596756

[39] Elsergany RN, Vreeman G, Sun CC. An approach for predicting the true density of powders based on in-die compression data. Int J Pharm. 2023;637:122875. 10.1016/j.ijpharm.2023.122875. 36948478 10.1016/j.ijpharm.2023.122875

[40] Six K, Leuner C, Dressman J, Verreck G, Peeters J, Blaton N, et al. T hermal properties of hot-stage extrudates of itraconazole and eudragit E100 phase separation and polymorphism. J Therm Anal Calorim. 2002;68:591–601. 10.1023/A:1016056222881.

[41] Porat D, Dukhno O, Vainer E, Cvijić S, Dahan A. Antiallergic treatment of bariatric patients: potentially hampered solubility/dissolution and bioavailability of loratadine, but not desloratadine, post-bariatric surgery. Mol Pharm. 2022;19:2922–36. 10.1021/acs.molpharmaceut.2c00292. 35759355 10.1021/acs.molpharmaceut.2c00292

[42] Tung Hiep B, Ois Gimenez F, Khanh V, Kim Hung N, Thuillier A, Farinotti R, et al. Binding of chlorpheniramine enantiomers to human plasma proteins. Chirality. 1999;11(5–6):501–4. 10.1002/(SICI)1520-636X(1999)11:5/6<501::AID-CHIR24>3.0.CO;2-K. 10368923 10.1002/(SICI)1520-636X(1999)11:5/6<501::AID-CHIR24>3.0.CO;2-K

[43] Eckhart CG, McCorkle T. Chlorpheniramine maleate. Anal Profiles Drug Subst Excipients. 1978;43–80. 10.1016/S0099-5428(08)60089-1

[44] Kassis V, Søndergaard J. Heat-separation of normal human skin for epidermal and dermal prostaglandin analysis. Arch Dermatol Res. 1982;273:301–6. 10.1007/BF00409259. 6762159 10.1007/BF00409259

[45] Kinvig H, Cottura N, Lloyd A, Frivold C, Mistilis J, Jarrahian C, et al. Evaluating islatravir administered via microneedle array patch for long-acting HIV pre-exposure prophylaxis using physiologically based pharmacokinetic modelling. Eur J Drug Metab Pharmacokinet. 2022;47:855–68. 10.1007/s13318-022-00793-6. 36178586 10.1007/s13318-022-00793-6 PMC9744694

[46] Davidson A, Al-Qallaf B, Das DB. Transdermal drug delivery by coated microneedles: Geometry effects on effective skin thickness and drug permeability. Chem Eng Res Des. 2008;86:196–206. 10.1016/j.cherd.2008.06.002.

[47] Makvandi P, Kirkby M, Hutton AR, Shabani M, Yiu CK, Baghbantaraghdari Z, et al. Engineering microneedle patches for improved penetration: analysis, skin models and factors affecting needle insertion. Nano-Micro Lett. 2021;13(1):93. 10.1007/s40820-021-00611-9. 10.1007/s40820-021-00611-9 PMC8006208 34138349

[48] Donnelly RF, Garland MJ, Morrow DIJ, Migalska K, Singh TRR, Majithiya R, et al. Optical coherence tomography is a valuable tool in the study of the effects of microneedle geometry on skin penetration characteristics and in-skin dissolution. J Control Release. 2010;147:333–41. 10.1016/j.jconrel.2010.08.008. 20727929 10.1016/j.jconrel.2010.08.008

[49] Kochhar JS, Quek TC, Soon WJ, Choi J, Zou S, Kang L. Effect of microneedle geometry and supporting substrate on microneedle array penetration into skin. J Pharm Sci. 2013;102:4100–8. 10.1002/jps.23724. 24027112 10.1002/jps.23724

[50] Paredes AJ, Volpe-Zanutto F, Permana AD, Murphy AJ, Picco CJ, Vora LK, et al. Novel tip-loaded dissolving and implantable microneedle array patches for sustained release of finasteride. Int J Pharm. 2021;606:120885. 10.1016/j.ijpharm.2021.120885. 34271153 10.1016/j.ijpharm.2021.120885

[51] Catlin EJ, Lopez-Vidal L, Donnelly RF, Paredes AJ. A dynamic duo comes of age: nanocrystals and microneedles for hydrophobic drug delivery. Expert Opin Drug Deliv. 2025;14:1–8. 10.1080/17425247.2025.2531059. 10.1080/17425247.2025.2531059 40625242

[52] Khiao In M, Richardson KC, Loewa A, Hedtrich S, Kaessmeyer S, Plendl J. Histological and functional comparisons of four anatomical regions of porcine skin with human abdominal skin. Anat Histol Embryol. 2019;48:207–17. 10.1111/ahe.12425. 30648762 10.1111/ahe.12425

[53] Heykants J, Van Peer A, Van De Velde V, Van Rooy’ P, Meuldermans W, Lavrijsen K, et al. The clinical pharmacokinetics of itraconazole: an overview. Mycoses. 1989;32:67–87. 10.1111/j.1439-0507.1989.tb02296.x. 2561187 10.1111/j.1439-0507.1989.tb02296.x

[54] Shebley M, Sandhu P, Emami Riedmaier A, Jamei M, Narayanan R, Patel A, et al. Physiologically based pharmacokinetic model qualification and reporting procedures for regulatory submissions: a consortium perspective. Clin Pharmacol. 2018;104(1):88–110. 10.1002/cpt.1013. 10.1002/cpt.1013 PMC6032820 29315504

[55] Rajoli RKR, Flexner C, Chiong J, Owen A, Donnelly RF, Larrañeta E, et al. Modelling the intradermal delivery of microneedle array patches for long-acting antiretrovirals using PBPK. Eur J Pharm Biopharm. 2019;144:101–9. 10.1016/j.ejpb.2019.09.011. 31525446 10.1016/j.ejpb.2019.09.011 PMC6917207

[56] Kinvig H, Rajoli RKR, Pertinez H, Vora LK, Volpe-Zanutto F, Donnelly RF, et al. Physiologically based pharmacokinetic modelling of cabotegravir microarray patches in rats and humans. Pharmaceutics. 2023;15(12):2709. 10.3390/pharmaceutics15122709. 38140050 10.3390/pharmaceutics15122709 PMC10747499

