# Supplementary material for: From simulation to application: enhancing preclinical evaluation of dissolvable microarray patches through PBPK modelling
Source: Drug Deliv Transl Res. 2025 Oct 10;16(5):1592–606. doi: 10.1007/s13346-025-01974-x (PMC13038671; doi:10.1007/s13346-025-01974-x)
Supplement: Supplementary file 1 — Supplementary file1 (DOCX 484 KB) [file 13346_2025_1974_MOESM1_ESM.docx]

**Supplementary Information**

**From Simulation to Application:  Enhancing Preclinical Evaluation of Dissolvable Microarray Patches Through PBPK modelling**

Maja Railic*^a^, Wilhelmus E. A. de Witte^b^, Stephan Schaller^b^, Sarah Toluwanimi Agboola^c^, [Ziad Sartawi](https://advanced.onlinelibrary.wiley.com/authored-by/Sartawi/Ziad) ^d^, Waleed Faisal ^d^, Mohamed Elkhashab ^a^, Abina Crean^a^, Sonja Vucen^a^

^a^ SSPC, Research Ireland Centre for Pharmaceuticals, School of Pharmacy, University College Cork, Cork T12 K8AF, Ireland

^b^ ESQlabs GmbH, Am Sportplatz 7, Saterland 26683, Germany

^c^ School of Pharmacy, University College Cork, Cork T12 K8AF, Ireland

^d^ ArrayPatch Ltd., Euro Business Park, Little Island, Cork T45 FX94, Ireland

*Corresponding author

SSPC, Research Ireland Centre for Pharmaceuticals, School of Pharmacy, University College Cork, Cork T12 K8AF, Ireland

Email: 121100335@umail.ucc.ie

**Table S1**: List of parameters underwent sensitivity analysis, their corresponding descriptions and equations (where applicable). Equations are adapted from Dancik *et al* (1).

*Abbreviations:* $\varphi_{a}$ *- fraction of aqueous phase accessible to albumin;* $f_{u}$*- fraction of compound in the viable tissues that is unbound to protein;* $\varphi_{ilp}$*- lipid volume fraction of the aqueous phase;* $f_{non/vt}$*- fraction of non-ionizable solute in the viable tissues;* $K_{o/w}$*-lipophilicity;* $D_{free}$*- diffusion of unbound molecules in viable epidermis and dermis;* $D_{bound}$*-diffusion of molecule bound to protein;* $P_{sc/w}$*- permeability coefficient in stratum corneum relative to water;* $K_{sc/w}$ *– partition coefficient stratum corneum-water;* $\varphi_{aq}$*- aqueous volume fraction (totally accessible to solutes);* $K_{lip/w}$*- partition coefficient lipid phase-water;* $\varphi_{cor}$*- corneocyte-phase volume fraction;* $K_{cor/w}$ *– partition coefficient corneocyte-water; n/a - not applicable.*

| **Parameter** | **Description** | **Equation** |
| --- | --- | --- |
| Binding factor | Correction factor for binding to skin proteins | $Binding factor=\left( 1-\varphi_{a} \right)+\varphi_{a}/f_{u}+\varphi_{ilp}f_{non/vt}K_{o/w}$ |
| D_ED_ | Diffusion coefficient of drug in epidermis | $D_{ed}=(D_{free}+\varphi_{a} D_{bound} (1-fu )/f_{u}) /Binding factor$ |
| D_DE_ | Diffusion coefficient of drug in dermis | $D_{de}=(D_{free}+\varphi_{a} D_{bound} (1-fu )/f_{u}) /Binding factor$ |
| D_sc_ | Diffusion coefficient of drug in stratum corneum | $D_{sc}=(P_{sc/w}h_{sc}) /K_{sc/w}$ |
| h_DE_ | Dermis thickness | n/a |
| h_ED_ | Epidermis thickness | n/a |
| K_DE_ | Drug’s partition coefficient in dermis | $K_{de}=(\varphi_{aq}/f_{non/vt})* Binding factor$ |
| K_ED_ | Drug’s partition coefficient in epidermis | $K_{ed}=(\varphi_{aq}/f_{non/vt})* Binding factor$ |
| K_SC_ | Drug’s partition coefficient in stratum corneum | $K_{sc}=\varphi_{lip}K_{lip/w}+\varphi_{cor}K_{cor/w}$ |
| h_SC_ | Stratum corneum thickness | n/a |

**Fig.S1**: Insertion profiles of (a) CPM, (b) LOR, and (c) ITZ MAP into Parafilm M® layers (n = 3). All tested MAP penetrated the third Parafilm M® layer, indicating an insertion depth of approximately 70% of the microneedle length. Y-error bars represent the standard deviation.

**Fig.S2**: Predicted vs observed LOR distribution in skin layers and release medium over time before optimisation of highly sensitive parameters: (a) epidermis, (b) dermis, and (c) cumulative release in the *in vitro* release medium (n = 2). Solid lines represent model predictions, dots indicate observed *in vitro* data, and the shaded region denotes the two-fold error margin for predicted values. Y-error bars represent the standard deviation.

**Fig.S3**: Predicted vs observed CPM distribution in skin layers and release medium over time before optimisation of highly sensitive parameters: (a) epidermis, (b) dermis, and (c) cumulative release in the in vitro release medium (n = 2). Solid lines represent model predictions, dots indicate observed *in vitro* data, and the shaded region denotes the two-fold error margin for predicted values. Y-error bars represent the standard deviation.

**Fig.S4**: Predicted vs observed ITZ plasma concentration over time before optimisation of highly sensitive parameters. Solid lines represent model predictions, dots indicate observed *in vivo* data, and the shaded region denotes the two-fold error margin for predicted values. Y-error bars represent the standard deviation.

**References**

*1. Dancik Y, Miller MA, Jaworska J, Kasting GB. Design and performance of a spreadsheet-based model for estimating bioavailability of chemicals from dermal exposure. Vol. 65, Advanced Drug Delivery Reviews. 2013. p. 221–36*
